# Association of anemia with worsened activities of daily living and health-related quality of life scores derived from the Minimum Data Set in long-term care residents

**DOI:** 10.1186/1477-7525-10-129

**Published:** 2012-10-19

**Authors:** Robert A Bailey, Gregory Reardon, Michael R Wasserman, R Scott McKenzie, R Steve Hord

**Affiliations:** 1 Janssen Scientific Affairs, LLC, Horsham, PA, USA; 2 Informagenics, LLC & The Ohio State University College of Pharmacy, Columbus, OH, USA; 3 Senior Care of Colorado, Aurora, CO, USA; 4 SAVA Senior Care, Tucker, GA, USA; 5 450 W. Wilson Bridge Rd., Suite 340, Worthington, OH, 43085, USA

**Keywords:** Anemia, Long-term care, Activities of daily living, Quality of life, Health status

## Abstract

**Background:**

Among long-term care (LTC) residents, we explored the association between anemia status and hemoglobin (Hb) level with Activities of Daily Living (ADL) functioning and health-related quality of life (HRQOL).

**Methods:**

Data were derived from the AnalytiCare database, containing laboratory and Minimum Data Set (MDS) reports for 27 LTC facilities in Colorado. Study timeframe was 1/1/07-9/15/08. Patients were selected based on: residence in LTC >90 days, Hb and serum creatinine value within 90 days of the earliest non-admission (index) MDS. From the index MDS, the method of 1) Carpenter et al. [BMC Geriatrics 6:7(2006)] was used to derive a summary measure of ADL performance (the MDS-ADL score) and 2) Wodchis et al. [IJTAHC 19:3(2003)] was used to assign HRQOL scores (MDS items were mapped to the Health Utilities Index Mark 2 (HUI2) scoring function to create the MDS-HSI score). Anemia was defined as Hb <12 g/dL females and <13 g/dL males. Adjusted linear regression was used to evaluate the independent association of anemia and hemoglobin level on MDS-ADL and MDS-HSI scores.

**Results:**

838 residents met all inclusion criteria; 46% of residents were anemic. Mean (SD) MDS-ADL score was 14.9 (7.5) [0–28 scale, where higher score indicates worse functioning]. In the adjusted model, anemia was associated with a significantly worse MDS-ADL score (+1.62 points, *P*=.001). Residents with Hb levels 10 to <11 g/dL had significantly worse ADL score (+2.06 points, *P*=.005) than the >13 g/dL reference. The mean MDS-HSI score was 0.431 (0.169) [range, where 0=dead to 1=perfect health]. Compared with non-anemic residents, in this adjusted model, residents with anemia had significantly worse MDS-HSI scores (−0.034 points, *P*=.005). Residents with hemoglobin levels <10 g/dL had significantly worse MDS-HSI scores (−0.058 points, *P*=.016) than the >13 g/dL reference.

**Conclusions:**

After adjusting for several covariates, LTC residents with anemia, and many of those with moderate to severe declines in Hb level, had significantly poorer outcomes in both ADL functioning and HRQOL. The association between Hb level and the HRQOL measure of MDS-HSI appears to be largely explained by the mobility domain of the HRQOL measure.

## Background

The prevalence of anemia is high among residents of long-term care (LTC) facilities [1]. Using the WHO definition [2] (hemoglobin [Hb] <12 g/dL for females and <13 g/dL for males) to define anemia status, the prevalence rate reported in five LTC studies has ranged from 40% to 60% [3-7].

Adverse clinical outcomes have been associated with anemia in the LTC setting. In studies that have included LTC residents, anemia has been linked to higher rates of falls [3,8,9] and hospitalizations [9,10]. Anemia in LTC residents has also been associated with a 17% higher risk of 1-year mortality (though not independently of activities of daily living (ADL) performance) [11], with a 60% higher risk of mortality among residents who later experienced a hip fracture [12], and with twice the risk of mortality in female residents [13].

Balducci et al. [14] hypothesize that elderly people generally have a reduced capacity to cope with illness, and therefore, in this population, health-related quality of life (HRQOL) and functional ability may be affected by declining health and anemia. In the elderly, anemia has been linked to deficits in physical performance [15-20], including functional decline in activities of daily living (ADL), though only one study has evaluated this association in the LTC setting. In a retrospective review of Minimum Data Set (MDS) assessments among 24 U.S. LTC facilities, Schnelle et al. found that anemic residents with chronic kidney disease (CKD) had significantly worse performance than non-anemic CKD residents on seven of ten individual ADL items [21].

Studies of older patients have also demonstrated significant associations between anemia [22-24] and HRQOL, though only one study has evaluated this association in LTC. In a study of LTC residents in Ontario, Lam et al. [25] found that anemia had a significant impact on HRQOL score, when applying an adjusted model that included 60 diseases that were also tested.

Given the high prevalence of anemia in LTC and its observed associations with specific clinical events, the current study evaluates in LTC residents the association of anemia and Hb level with a physical functioning index, the Carpenter et al. ADL score [26], and with an HRQOL index, the Minimum Data Set Health-Status Index (MDS-HSI) [27]. Both assessment measures are derived from the MDS, a comprehensive and longitudinal clinical assessment of LTC residents that is routinely collected in the U.S., Canada, and in more than 20 countries [26].

## Methods

### Study design

This study used a retrospective cross-sectional design. Data was obtained from the AnalytiCare LTC database for residents of 27 LTC facilities in the state of Colorado that contributed Minimum Data Set (MDS) version 2.0, laboratory records and pharmacy dispensing records to this integrated database. Although the MDS is a component of the medical chart in LTC facilities, data collected in this instrument may not capture all comorbid conditions noted elsewhere in the chart. The MDS 2.0 has been reported to have moderate to moderate/high validity and reliability, but may underreport some conditions such as depression [28]. Wodchis et al. reported sensitivities exceeding 0.60-0.80 for 22 common diagnoses, but also noted limitations in capturing several conditions [29]. Electronic MDS, laboratory and pharmacy records were complete for all included study residents, though use was not captured of over-the-counter medications not otherwise dispensed by LTC pharmacies. All data used in this study was de-identified, HIPAA compliant, and exempt from requirement for IRB review.

Data elements were collected for residents during the study timeframe of 1/1/07-9/15/08. To be included in this study, residents were required to have: 1) complete MDS, pharmacy and lab data available in the AnalytiCare database and at least one non-admission/non-discharge MDS assessment performed during the study timeframe, 2) residence in the LTC facility for more than 90 days (i.e. were not newly admitted to the LTC facility), 3) a hemoglobin (Hb) and serum creatinine (sCr) lab value within 90 days of the earliest non-admission MDS, and 4) documented age, gender, and race. The earliest periodic MDS assessment available during the study timeframe was identified as the *index MDS*. Residents were excluded from analysis if, on the index MDS, they were identified either to have a diagnosis of cancer, were receiving chemotherapy, were receiving renal dialysis, had end-stage renal disease, had a life expectancy of less than 6 months or were receiving hospice care.

### Anemia status and covariates

Anemia status was based solely on the Hb value (*index Hb*) of the lab test performed closest to, and within 90 days, of the index MDS date. *Anemia status* was defined using the World Health Organization definition [2] (where anemia occurs if Hb <12 g/dL for females and <13 for males). An alternative to anemia status, *hemoglobin range*, was estimated for each resident by assigning the index Hb to one of five values: ≥13 g/dL, 12 to <13, 11 to <12, 10 to <11, <10 [9]. Estimation of CKD, a covariate in the analysis, was identified from lab or MDS evidence if, from the sCr closest to and within 90 days of the MDS, the resident had either estimated GFR <60 mL/min/1.73 m^2^ (Modification of Diet in Renal Disease equation version 4) [30], or if the resident had renal failure checked on the index MDS Section I1, or else had an entry for chronic kidney disease recorded in MDS Section I3 *Other Current of More Detailed Diagnoses and ICD9 codes*. Use of certain prescription classes (also used as covariates in the analysis) were based on evaluation of all prescriptions dispensed 30 days prior to and 90 days after the index Hb. All other model covariates and the ADL and HRQOL endpoint measures were derived from the index MDS.

### MDS-ADL score

The ADL self-performance ratings in section G of the MDS version 2.0 have been used to evaluate physical functioning in the LTC facility [26,31]. Carpenter et al. [26] developed a method for estimating ADL performance in the LTC facility by summing the self-performance ratings for seven items in the MDS: bed mobility, transfer, locomotion, dressing, eating, toilet use and personal hygiene. Each item is rated by the LTC coordinator on a scale from 0 (independent) to 4 (total dependence). Alternatively, the coordinator is permitted to assign a value of “activity did not occur during the entire 7 days” to any ADL assessment item. Here, following Carpenter et al. [26] we assigned a substitute rating of 4 (total dependence) by assuming that the resident was incapable of self-performance on this ADL item during the seven-day recall period for this section. We then summed, for each resident, the value of the numeric rating for all seven items, calculating the total MDS-ADL^a^ performance score ranging from 0 (completely independent) to 28 (completely dependent). Thus a higher MDS-ADL score indicates worse ADL performance.

### MDS-HSI score

The HRQOL^b^ score was calculated by using the MDS-HSI methodology developed by Wodchis et al [27]. These authors developed this method as a practical means of mapping selected items from the standard MDS assessments conducted in LTC facilities and home care settings to an established measure of HRQOL, the Health Utilities Index Mark 2 [HUI2] [32,33]. The result of this approach yields both an overall HRQOL score, the MDS-HSI, and individual subscores for the six dimensions comprising the HUI2: sensation, mobility, emotion, cognition, self-care, and pain.

Wodchis et al. [27] selected the HUI2 as the basis for the MDS-HSI score, rather than the newer HUI3 instrument, since the HUI2 permits a focus on self-care, considers pain in the context of analgesics to manage it, and places emotion in the context of worry and anxiety rather than happiness vs. depression. The HUI instruments have been administered as both self- and proxy-assessments (e.g. by a spouse or healthcare professional on behalf of the responsible individual) [33]. In their original work, Wodchis et al. [27] found preliminary evidence of convergent and construct validity for the MDS-HSI by first, comparing single-attribute scores from the MDS-HSI with summated scales based on the same attributes, and secondly by separately comparing MDS-HSI scores between residents of supportive housing, recipients of home care services in the community, LTC residents, and patients in a chronic care hospital with HUI2 scores obtained separately from an external reference population (1996 National Population and Health Survey) of community- and institutional-based respondents. A later study [34] assessed criterion validity of the MDS-HSI in a nursing home population against scores derived from interviewer-administered HUI2 in study residents. Wodchis et al. found analogous group-level concordance, but only moderate individual-level agreement. These authors concluded that the MDS-HSI can be used to substitute for the HUI2 in group-level comparisons but not for individual clinical evaluation comparisons [34].

Following the MDS-HSI to HUI2 mapping algorithm reported by Wodchis et al. [27], we calculated, from the index MDS, for each study resident a summary MDS-HSI score and individual subscores for the six MDS-HSI dimensions. The summary score is interpreted such that a value of 1 represents the preference assigned perfect health, while a value of 0 represents dead.

### Statistical analysis

Stata (Intercooled 8.0, College Station TX) was used to conduct statistical analysis. Unadjusted estimates of MDS-ADL and MDS-HSI scores were compared through cross tabulation with both anemia status and hemoglobin range. Multiple regression, adjusted for covariates potentially related to the study endpoints (demographics, renal function, falling history, conditions; a full listing of covariates is shown in Tables 1 and 2), was conducted to separately evaluate the potential independent effect of 1) anemia status, and 2) Hb range, on the study endpoints of MDS-ADL and MDS-HSI scores. Starting with the large set of covariates, backward elimination was used to efficiently reduce the final set of retained covariates, retaining those covariates with *P*≤0.25 through each successive iteration of the model. Since the WHO definition of anemia incorporates gender with Hb to determine anemia status, the interaction term of gender with anemia status was added to the anemia models.

**Table 1:** Resident demographics and hemoglobin

	**Not anemic**	**Anemic**	**p-value**^†^	**All residents**
	***n***	***n***		**n**
	**452**	**386**		**838**
*Demographics*				
Age (years)				
Median [interquartile range]	81 [71–88]	82 [72–88]	-	82 [72–88]
≤65	17%	15%	0.627	16%
65-74	13%	15%		14%
75-84	31%	31%		31%
85+	39%	39%		39%
Female	74%	59%	<0.001	67%
Race/Ethnicity				
White, not of Hispanic Origin	81%	82%	0.472	82%
Black, not of Hispanic Origin	4%	5%		5%
Asian/Pacific Islander	2%	1%		2%
Hispanic	12%	12%		12%
Am Indian/Alaskan Native	0%	0%		0%
Other	0%	0%		0%
Educational Level				
Less than 12 years	18%	23%	0.260	20%
High school graduate	39%	34%		37%
Some college	14%	17%		15%
College graduate	10%	10%		10%
Unknown	19%	17%		18%
*Hemoglobin*				
Mean (SD) Hemoglobin Level (g/dL)	13.6 (1.1)	10.9 (1.0)	<0.001	12.4 (1.7)
Anemic (from index Hb, WHO definition)*	0%	100%	<0.001	46%
Hemoglobin Level ≥13 g/dL	68%	0%	<0.001	37%
Hemoglobin Level 12 to <13	32%	12%		23%
Hemoglobin Level 11 to <12	0%	41%		19%
Hemoglobin Level 10 to <11	0%	31%		14%
Hemoglobin Level <10	0%	16%		8%

Terms: WHO, World Health Organization.

* WHO definition of anemia = index Hb <12 g/dL if female, <13 g/dL if male.

^†^ F-tests used for differences in means; Chi-square tests used for proportions.

**Table 2:** Resident conditions

	**Not anemic**	**Anemic**	**p-value**^†^	**All residents**
	***n***	***n***		**n**
	**452**	**386**		**838**
*Renal Function*				
Section I CKD or GFR Stage 3-5	37%	52%	<0.001	44%
*Falling History*				
Fell in Past 30 or 180 Days	45%	48%	0.377	47%
*Diseases and Conditions*				
Alzheimer's disease	9%	3%	0.002	6%
Arteriosclerotic heart disease	2%	6%	0.009	4%
Arthritis	11%	10%	0.713	10%
Asthma	2%	2%	0.889	2%
Bone Fracture (Pathological)	2%	4%	0.114	3%
Cerebral Palsy	0%	0%	0.355	0%
Cerebrovascular Accident	15%	16%	0.762	15%
Congestive Heart Failure	10%	14%	0.089	12%
Emphysema/COPD	7%	15%	<0.001	10%
Dementia (not Alzheimer's)	23%	24%	0.769	24%
Deep Vein Thrombosis	1%	2%	0.149	1%
Diabetes Mellitus	25%	37%	<0.001	30%
Hemiplegia/hemiparesis	6%	5%	0.332	5%
Hip Fracture	2%	5%	0.004	3%
Hypotension	4%	2%	0.098	3%
Missing Limb	1%	1%	0.846	1%
Multiple Sclerosis	4%	2%	0.051	3%
Osteoporosis	7%	7%	0.962	7%
Peripheral Vascular Disease	4%	4%	0.649	4%
Paraplegia	0%	1%	0.874	0%
Parkinson's Disease	1%	1%	0.566	1%
Quadriplegia	1%	1%	0.349	1%
Seizure Disorder	6%	3%	0.134	5%
Transient Ischemic Attack	1%	1%	0.864	1%
*Current Medications*				
Antianxiety/Hynotic/ Antipsychotic	82%	80%	0.625	81%
Antidepressant	67%	67%	0.898	67%
Beta Blocker	27%	43%	<0.001	35%
Diuretic	41%	49%	0.023	45%

All values were obtained from the index MDS, index hemoglobin, serum creatinine or the pharmacy database (with fill dates 30 days prior to 90 days after the index hemoglobin). ^†^Chi-square tests used for proportions.

## Results

Of the 1,460 residents who satisfied inclusion rules, 622 who met also met one or more exclusion rules, thus leaving 838 eligible residents for analysis. Summary statistics are shown in Table 1 (demographics and hemoglobin) and Table 2 (conditions). The median resident age was 82 years (resident ages in the study database were truncated at 90 years due to HIPAA safe-harbor rules), and the age distribution was similar between the anemic and non-anemic groups. Index Hb levels were lower on average by 2.7 g/dL for the 46% of residents with lab-defined anemia than for the 54% who did not have anemia (*P*<.001). A lower proportion of anemic residents (59%) were female compared with non-anemic ones (74%, *P*<.001, Chi-square). The distribution of race/ethnicity categories was similar among anemic and non-anemic groups. Distribution by Hb range revealed that approximately 40% of all residents had Hb levels below 12 g/dL (Table 1).

Of the 838 study residents, all had complete data for the seven self-performance MDS items required to calculate an MDS-ADL score and were included in the ADL analysis. Unadjusted analysis revealed that the mean (SD) MDS-ADL score for all study residents was 14.9 (7.5) [of 0 to 28 possible range where a lower score means better ADL performance]. Mean (SD) MDS-ADL score for the non-anemic population was 14.3 (8.0) and for the anemic population was 15.5 (6.8) [*P*=.014, t-test].

Table 3 shows findings from the adjusted regression model for anemia status and MDS-ADL score. Anemia was associated with a 1.62 worse ADL score when compared with the non-anemic reference case (*P*=.001). Of the covariates retained within the model, a significantly poorer MDS-ADL score was seen, in descending order of association with, cerebral palsy, quadriplegia, multiple sclerosis, age 85+, Asian/Pacific islander, age 75–84, hemiplegia, age 65–74, cerebral vascular accident, Alzheimer’s disease, female, tech school/some college education, fell within 180 days prior to the index MDS, and Section I CKD or GFR Stage 3–5. The latter had a negative association with poorer MDS-ADL score.

**Table 3:** Regression model for anemia status and MDS-ADL score

	**Coefficient**	**95% Lower bound**	**95% Upper bound**	**p-value**
Anemic (Index Hb)	1.62	0.63	2.62	0.001
Female	1.66	0.60	2.71	0.002
Age 65-74	2.94	1.17	4.72	0.001
Age 75-84	4.00	2.41	5.58	<0.001
Age 85+	4.57	2.96	6.18	<0.001
Black, not of Hispanic Origin	2.18	−0.17	4.53	0.069
Asian/Pacific Islander	4.01	0.27	7.75	0.036
Tech School or Some College	1.50	0.16	2.84	0.029
College Grad or Advanced Degree	1.45	−0.15	3.04	0.075
Section I CKD or GFR Stage 3-5	−1.20	−2.22	−0.18	0.022
Fell in Past 180 Days	1.30	0.34	2.26	0.008
Alzheimer's Disease	2.52	0.52	4.51	0.014
Bone Fracture	2.03	−0.66	4.71	0.139
Cerebral Palsy	18.87	5.06	32.68	0.007
Cerebral Vascular Accident	2.65	1.18	4.13	<0.001
COPD	1.43	−0.16	3.02	0.078
Diabetes Mellitus	0.75	−0.33	1.84	0.174
Hemiplegia	3.78	1.45	6.12	0.002
Multiple Sclerosis	7.54	4.51	10.56	<0.001
Parkinson's Disease	3.65	−0.99	8.30	0.123
Quadriplegia	9.90	4.92	14.87	<0.001
Seizure	1.78	−0.58	4.13	0.139
Transient Ischemic Attack	−4.06	−9.35	1.23	0.132
Antianxiety/Hynotic/Antipsychotic Med	−1.28	−2.96	0.41	0.138
Antidepressant Medication	0.85	−0.55	2.25	0.234
Intercept	7.78	5.82	9.75	<0.001

MDS-ADL Score (0–28 where higher score is worse score). Reference is not anemic, male, age <65 years, white race, not completed high school, GFR stage 1–2 (from serum creatinine) or no MDS Section I CKD noted, not fallen in past 180 days, absence of conditions or drugs listed. n=838. Adjusted r^2^=0.1454.

Table 4 shows findings from the sensitivity analysis for the MDS-ADL and MDS-HSI adjusted regression models, where Hb range is substituted for anemia status, and the interaction term of anemia with gender is removed. For the MDS-ADL, when compared with the reference range of Hb ≥13 g/dL, residents with Hb levels 11 to <12 (+0.85 points, *P*=.184), 10 to <11 (+2.06 points, *P*=.005) and <10 (+1.79 points, *P*=.059) had worse MDS-ADL scores, though only the difference for Hb 10 to <11 was significant. A coefficient for Hb level 12 to <13 is not shown in Table 4 since this Hb level was removed by the regression model during the backwards selection process, having a *P*-value exceeding 0.25.

**Table 4:** Sensitivity analysis: Regression models for hemoglobin range with MDS-ADL score and MDS-HSI score

	**Coefficient**	**95% Lower bound**	**95% Upper bound**	**p-value**
**MDS-ADL score**				
Hemoglobin Level 11 to <12 g/dL	0.85	−0.40	2.10	0.184
Hemoglobin Level 10 to <11	2.06	0.62	3.50	0.005
Hemoglobin Level <10	1.79	−0.07	3.65	0.059
**MDS-HSI score**				
Hemoglobin Level 12 to <13 g/dL	−0.026	−0.056	0.005	0.101
Hemoglobin Level 11 to <12	−0.021	−0.053	0.012	0.209
Hemoglobin Level 10 to <11	−0.031	−0.067	0.006	0.099
Hemoglobin Level <10	−0.058	−0.104	−0.011	0.016

MDS-ADL Score (0–28 where higher score is worse score). MDS-HSI Score where 0=dead and 1=perfect health. Reference is hemoglobin ≥13 g/dL. Findings from these adjusted models show only estimates for hemoglobin ranges in this table, and only for those ranges that were retained in the final models after backwards elimination (*P*<0.25).

**Figure 1:**
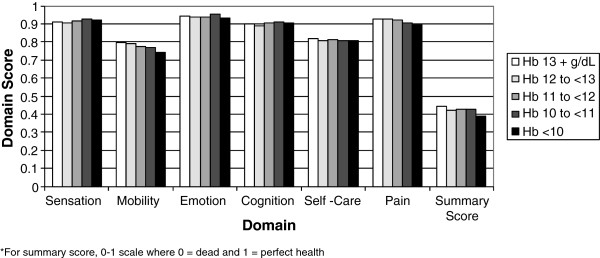
Mean MDS-HSI score by hemoglobin range.

For estimation of MDS-HSI scores, 777 of the 838 retained residents had non-missing data for all MDS elements required to calculate a summary MDS-HSI score and were included in the MDS-HSI analysis. In this unadjusted analysis, mean (SD) MDS-HSI score for all study residents was 0.431 (0.169) [of 0 to 1 possible range where a higher score means a better HRQOL]. Mean (SD) MDS-HSI score for the non-anemic population was 0.442 (0.175) and for the anemic population was 0.418 (0.161) [*P*=.048, t-test]. Figure 1 shows the distribution of both the mean MDS-HSI summary scores for each of the five categories of Hb range, as well as the mean individual domain scores.

In the adjusted regression model for MDS-HSI score (Table 5) having anemia was associated with a 0.034 lower or worse MDS-HSI score when compared with the non-anemic reference case (*P*=.005). Of the covariates retained within the model, a significantly worse MDS-HSI summary score was seen, in descending order of association with quadriplegia, multiple sclerosis, Asian/Pacific Islander, age 65–74, Alzheimer's disease, age 85+, age 75–84, hemiplegia, fell within 180 days prior to the index MDS, tech school/some college education, and female.

**Table 5:** Regression model for anemia status and MDS-HSI score

	**Coefficient**	**95% Lower bound**	**95% Upper bound**	**p-value**
Anemic (Index Hb)	−0.034	−0.057	−0.010	0.005
Female	−0.027	−0.052	−0.002	0.037
Age 65-74	−0.090	−0.133	−0.048	<0.001
Age 75-84	−0.065	−0.103	−0.028	0.001
Age 85+	−0.084	−0.122	−0.047	<0.001
Black, not of Hispanic Origin	−0.047	−0.103	0.008	0.096
Asian/Pacific Islander	−0.099	−0.195	−0.003	0.044
Tech School or Some College	−0.037	−0.068	−0.006	0.021
Section I CKD or GFR Stage 3-5	0.018	−0.006	0.042	0.142
Fell in Past 180 Days	−0.051	−0.074	−0.028	<0.001
Alzheimer's Disease	−0.094	−0.142	−0.047	<0.001
Bone Fracture	−0.058	−0.120	0.003	0.064
Cerebral Palsy	−0.244	−0.562	0.074	0.133
Cerebral Vascular Accident	−0.026	−0.060	0.009	0.145
Hemiplegia	−0.063	−0.119	−0.007	0.026
Multiple Sclerosis	−0.122	−0.193	−0.051	0.001
Parkinson's Disease	−0.094	−0.199	0.011	0.081
Quadriplegia	−0.191	−0.306	−0.077	0.001
Seizure	−0.044	−0.100	0.011	0.119
Antidepressant Medication	−0.015	−0.040	0.009	0.221
Intercept	0.589	0.549	0.630	<0.001

MDS-HSI Score where 0=dead and 1=perfect health. Reference is not anemic, male, age <65 years, white race, not completed high school, GFR stage 1–2 (from serum creatinine) or no MDS Section I CKD noted, not fallen in past 180 days, absence of conditions or drugs listed. n=777. Adjusted r^2^=0.1155.

Table 4 shows findings of the sensitivity analysis for MDS-HSI adjusted regression model, where Hb range is substituted for anemia status, and the interaction term of anemia with gender is removed. Compared with the reference range of Hb ≥13 g/dL, the MDS-HSI summary score was worse for Hb 12 to <13 (−0.026 points, *P*=.101), 11 to <12 (−0.021 points, *P*=.209), 10 to <11 (−0.031 points, *P*=.099), and <10 (−0.058 points, *P*=0.016). Only the finding for Hb <10 g/dL was significant. Since four regression models were evaluated in this analysis (anemia with MDS-ADL, anemia with MDS-HSI, Hb range with MDS-ADL, Hb range with MDS-HSI) the familywise error rate was maintained as a further sensitivity test by recalculating the individual test type I error rate to α/n = 0.0125. In this case, significant findings for the anemia and Hb range associations were maintained, but with the exception of the significant MDS-HSI finding for Hb <10 g/dL (P=0.016), which exceeded this threshold.

## Discussion

In the past, anemia was viewed as a sign of underlying disease, while today this condition is considered by many to be a cause itself of severe deterioration of quality of life, morbidity, decline in physical function, and a risk factor for death [14]. Within the specific context of managing anemia in the elderly patient, Balducci [35] identifies the “compression of morbidity” as one of the most worthwhile goals of geriatric medicine. Here the goal of the clinician is to delay and reverse the manifestations of aging to improve function, quality of life, and overall well-being of the geriatric patient. Thomas [36] offers similar support for including functioning and quality endpoints in geriatrics, concluding that there is a direct and independent effect of hemoglobin concentration on the symptom scores used to assess quality of life in a number of disease conditions: “The data suggest that we may not be able to modify the course of chronic kidney disease or other chronic diseases, but we do have the capacity to ameliorate the symptom complex that frequently is associated with anemia” [36].

### Anemia and ADL

Performance indicators such as ADL may be relevant predictors of physiologic outcomes in their own right. Abicht-Swensen et al. [37] observed in LTC residents that ADL, as calculated from the MDS, was a strong independent predictor of short-term mortality. Van Dijk et al. [11] found that when ratings for four items in the ADL section of the MDS were added to an adjusted model, that ADL was significantly associated with mortality within the first year of an MDS assessment. This association was independent of anemia and other resident factors.

In the current study the mean MDS-ADL score was 15.5 for the anemic residents and 14.3 for non-anemic residents. This compares with a mean score of 14.8 among mildly cognitively impaired residents and a worsened 19.0 mean score among severely cognitively impaired residents within a single U.S. nursing home reported in Carpenter et al.’s original work on the MDS-ADL [26]. Regarding the potential association between anemia and poorer physical functioning, the evidence for such a link is strong. In the unadjusted analysis in the current study, the mean MDS-ADL summary score was significantly worse for anemic residents. After adjusting for potential confounders in the regression model, we found that anemia was independently associated with greater impairment of MDS-ADL performance. In the adjusted sensitivity analysis substituting Hb range for anemia status, we found that only the moderately severe anemia range of 10 to <11 g/dL had a significantly lower MDS-ADL score when compared with the >13 g/dL reference. The next lowest Hb range of <10 g/dL had a regression coefficient that was similar to the 10 to <11 g/dL range, but the former was not significant since many fewer residents had this severely low value (Table 1).

As described above, Schnelle et al. found in an unadjusted analysis of single-item ADL ratings, that anemic LTC residents with CKD had significantly worse performance than non-anemic CKD residents on every ADL item except for “locomotion off unit,” “eating” and “personal hygiene.” [21] Earlier studies have consistently reported an association between anemia and ADL function for older persons in the community [15-20].

### Anemia and HRQOL

The mean MDS-HSI score was 0.418 for the anemic cohort (0.411 for females, 0.428 for males) and 0.442 for non-anemic one (0.430 for females, 0.474 for males). Two studies of residents at hospital-based LTC facilities in Ontario have reported substantially lower MDS-HSI scores (0.31 for females, 0.38 for males) [27] and (0.293 for females, 0.286 for males) [25] The somewhat lower MDS-HSI scores found in these earlier LTC studies might be due to high levels of medical impairment noted among these hospital-based cohorts, though MDS-HSI scores from both the current and these earlier LTC studies were both far lower than comparable HUI2 normative scores for the U.S. community population of 0.85 (65–74 years) and 0.83 (75–89 years).

Findings from the current study suggest that there is an association between anemia and impaired quality of life. In the unadjusted analysis in the current study, the mean MDS-HSI summary score was significantly worse for anemic residents. Of the six domains comprising the MDS-HSI summary score, Figure 1 shows that declines in mobility with decreasing Hb level appear to explain much of this difference in quality-of-life assessment between anemic and non-anemic residents. We found, in our adjusted analysis, that anemia was independently associated with a worse MDS-HSI score. In the adjusted sensitivity analysis substituting Hb range for anemia status, we found that only the severe anemia range of <10 g/dL had a significantly lower MDS-HSI score when compared with the >13 g/dL reference.

Several earlier studies have evaluated the association between quality-of-life measures and anemia. As described above, Lam et al. found that anemia had a significant association with HRQOL in the LTC setting [25]. That study assessed HRQOL with the same MDS-HSI measure used in the current study, though in a different LTC population (hospital-based LTC facility in Ontario) and utilizing a different regression modeling approach (full model retaining 60 disease predictors). In that study anemia was significantly associated with only 0.022 unadjusted and 0.006 lower adjusted MDS-HSI score vs. the significantly lower scores of 0.024 (unadjusted) and 0.034 (adjusted) that we found in the current study of a pool of U.S. nursing homes and utilizing a stepwise regression model. Studies outside of the LTC setting have also reported an association of anemia with HRQOL in older patients [22-24].

### Evaluating clinically-meaningful difference

In the current study, the independent effect of anemia was a 1.62 point worse adjusted MDS-ADL score. Findings from this study also showed that anemia was associated with a 0.034 adjusted lower MDS-HSI summary score. When modeling relationships between a condition such as anemia and worsened performance on humanistic measures at a single point in time, one concern is interpretation of the meaning of such differences. A limitation of the current study is that one can only speculate regarding such meaning; meaningful differences are not formally assessed in such between-group models [38-40]. Minimally-important *individual change* differences have been assessed, but the latter are not directly comparable to between-group model effects. For instance, Carpenter et al. evaluated changes in the MDS-ADL within individuals and concluded that ”[a] change of one point in the MDS-ADL scale denotes a clinically meaningful change.” A difference of 0.03 for the Health Utilities Index (HUI) instruments has been identified by Drummond [41] and Grootendorst et al. [42] as a clinically-important change. Drummond [41] has stated that a difference as low as 0.01 may be may be meaningful and important in some settings.

### Limitations

This study has several additional limitations. First, even though the LTC facility coordinator conducting the MDS assessment is directed to observe and communicate with the resident when completing the assessment, data is gathered from multiple sources [43], thus assessments of MDS-ADL and MDS-HSI are largely by proxy, rather than by self-report, and so are dependent on the quality of these data sources. Second, although the MDS prompts the LTC facility coordinator to report, in detail, diseases and conditions currently experienced by the resident (either through checkbox or open-ended entry of conditions), underreporting of such diseases and conditions on the MDS has been known to occur [44]. However, other studies have found high inter-rater reliability of quality indicators and strong correlations with independent observation of residents [45,46]. Third, potential confounders with anemia in the adjusted regression models we were limited to only those data elements contained in the MDS, pharmacy fills or index lab values. Other relevant factors potentially related to the clinical endpoints studied may not have been included in this study. Fourth, the regression models only tested a linear association between anemia/hemoglobin range and the outcomes of MDS-ADL and MDS-HSI score. Non-linear regression models (e.g. quantile regression) or transformations of these scores might have revealed different findings. Finally, anemia status was determined by a single index Hb value, so we were unable to determine whether the anemia status was an acute or chronic state, what the cause of the anemia was, and whether the MDS-ADL and MDS-HSI assessments changed over time within residents as anemia status and Hb levels changed.

## Conclusions

After adjusting for several covariates, LTC residents who had anemia, and many of those who had moderate to severe declines from normal Hb levels, had significantly poorer outcomes in ADL functioning (MDS-ADL) and HRQOL (MDS-HSI). The association between Hb level and MDS-HSI is largely influenced by the relationship of Hb level with the mobility domain of this measure.

For the geriatric clinician, goals for managing anemia should include therapeutic efforts to improve hemoglobin levels among those who are severely anemic. Separate from this, a focus on improving related symptom complexes, including fatigue, lack of energy and functional capacity may itself directly benefit anemic patients [36]. Summary QOL and ADL scores, such as the ones evaluated here, can be readily electronically calculated for each resident from existing electronic MDS forms, which are completed at least once quarterly. Following the original intent of those researchers who designed or adapted these scoring algorithms [26,27] the MDS-ADL and MDS-QOL may find a role as meaningful, longitudinal clinical outcomes for other serious conditions besides anemia.

Further research is needed to test the association between changes in anemia status or Hb level against changes in MDS-ADL and MDS-HSI scores, longitudinally, within the same residents. Further research is also needed to further test and potentially improve the MDS-ADL and MDS-HSI scoring algorithms for use in LTC, including validation of the newly-introduced MDS version 3.0. For the MDS-ADL, research should test this measure against external instruments that objectively assess functional performance. Further research should also test the MDS-HSI against alternative measures of HRQOL applied to the same residents, and to assess content and construct validation of the MDS-HSI domains of sensation, mobility, emotion, cognition, self-care, and pain as adequate representation of HRQOL in LTC residents.

### Endnotes

^a^Carpenter et al. did not assign a name to their summary ADL measure. We introduce the term “MDS-ADL” here.

^b^Although some researchers regard health status and HRQOL as separate constructs, these terms are used interchangeably in the original work of Torrance et al. [Torrance GW, Feeny DH, Furlong WJ, Barr RD, Zhang Y, Wang Q. Multiattribute utility function for a comprehensive health status classification system. Health Utilities Index Mark 2. Med Care. Jul 1996;34(7):702–722].

## Abbreviations

LTC: Long-term care; Hb: Hemoglobin; ADL: Activities of daily living; CKD: Chronic kidney disease; MDS-HSI: Minimum Data Set Health Status Index; MDS: Minimum Data Set; MDS-ADL: Minimum Data Set Activities of Daily Living Index; sd: Standard deviation; HUI2: Health Utilities Index Mark 2; HUI3: Health Utilities Index Mark 3; HUI: Health Utilities Index; SF-36: SF-36 short form health survey.

## Competing interests

The current study was sponsored by Janssen Scientific Affairs, LLC. At the time this research was conducted RAB and RSM were employees of Janssen Scientific Affairs, LLC (a Johnson and Johnson company) and shareholders of Johnson and Johnson. MRW, GR, and RSH were consultants to Janssen Scientific Affairs, LLC (a Johnson and Johnson company). GR also received funding from Janssen Scientific Affairs, LLC for production of this manuscript.

## Authors’ contributions

RAB (study design/methodology, interpretation and manuscript writing), GR (study design/methodology, data analysis/interpretation and manuscript writing), MRW (study design, manuscript review/rewriting), RSM (study design, manuscript review/rewriting), RSH (study design, manuscript review/rewriting). All authors read and approved the final manuscript.
